# The Importance of Social Environment in Schizophrenia

**DOI:** 10.1192/j.eurpsy.2025.2248

**Published:** 2025-08-26

**Authors:** P. S. Pires, C. Cunha, R. Cabral, F. Cunha, I. Santos, A. P. Costa

**Affiliations:** 1 DPSM, ULS Viseu Dão-Lafões, Viseu, Portugal

## Abstract

**Introduction:**

Patients with schizophrenia spectrum disorders continue to face societal stigma. This stigma contributes to their loneliness and marginalization, acting as a significant barrier to recovery and clinical stabilization. While medication and clinical follow-up are essential for treatment, social factors are also crucial for individuals to achieve a functional and fulfilling life. It is necessary to explore these factors in this this study.

**Objectives:**

To explore the social factors affecting the recovery and clinical stabilization of patients with schizophrenia spectrum disorders.

- To identify the support needs of these patients to enhance their functional and fulfilling lives.
- To assess the impact of societal stigma on the well-being and integration of individuals with schizophrenia.
- To examine the role of mental health professionals and family involvement in reducing stigma and improving social functioning.
- To highlight the importance of vocational interventions and supportive environments in facilitating the integration of patients into society.

**Methods:**

For the purpose of conducting the non-systematic narrative review on the topic, we performed a search for articles in the PubMed database.

**Results:**

Improvements in the effectiveness of antipsychotics and earlier intervention are enabling more patients to reach a cognitive level that supports a functional life, including maintaining interpersonal and occupational relationships. Thematic analysis has identified four key support needs: skill development, vocational intervention, support and encouragement, and a supportive work environment.

The involvement of mental health professionals with the patient’s family is also crucial for addressing and reducing the stigma associated with the illness, thereby enhancing understanding of the individual within the context of their condition.

Social anhedonia, which impairs social functioning, is a significant concern. Additionally, the risk of suicide is notably higher during the initial phase of schizophrenia compared to the general population.

**Conclusions:**

After achieving clinical stabilization with antipsychotics and other psychotropic medications, intervention in the social sphere becomes crucial for the patient’s well-being and functionality. Having a professional occupation, when feasible, is a positive indicator of patients’ integration and their role in society.

To support this integration, psychiatry services and civil society must enhance their efforts. This includes developing occupational services, establishing partnerships with local businesses, and improving public awareness about schizophrenia.

**Disclosure of Interest:**

None Declared

